# The Neo-MILK Web App as a Health Technology to Support Mothers of Preterm and Sick Neonates During Lactation: Usability Study

**DOI:** 10.2196/69079

**Published:** 2025-07-09

**Authors:** Isabella Schwab, Tim Ohnhaeuser, Roxane Lee Rothe, Till Dresbach, Katharina Schmitz, Natalie Tutzer, Nicola Gabriela Dymek, Juliane Köberlein-Neu, Nadine Scholten, Neo-Milk

**Affiliations:** 1 Institute of Medical Sociology, Health Services Research, and Rehabilitation Science, Faculty of Medicine and University Hospital Cologne, University of Cologne, Eupener Straße 129, Cologne, 50933, Germany; 2 Department for Psychosomatic Medicine and Psychotherapy, Center for Health Communication and Health Services Research, University Hospital Bonn, Bonn, Germany; 3 Department of Neonatology and Pediatric Intensive Care, Children's Hospital, University of Bonn, Bonn, Germany; 4 Department of Neonatology, Center for Pediatric and Adolescent Medicine, University Medical Center of the Johannes Gutenberg-University Mainz, Mainz, Germany; 5 TAKEPART Media + Science GmbH, Cologne, Germany; 6 Centre for Health Economics and Health Services Research, University of Wuppertal, Wuppertal, Germany

**Keywords:** mHealth, lactation, usability, perceived usefulness, lactation web app, preterm and sick infants, pumping, mother’s own milk, mobile health

## Abstract

**Background:**

Mothers of sick and preterm infants need support to establish and maintain lactation. Although many health technologies on breastfeeding are available, most lack in evidence-based information and are therefore not appropriate for educating mothers. Furthermore, they do not focus on the special challenges of mother-infant separation during lactation in mothers of sick or preterm infants.

**Objective:**

The aim of this study is to examine the usability and perceived usefulness of the evidence-based information about lactation and documentation tools contained in the Neo-MILK web app.

**Methods:**

A cross-sectional online survey was conducted among mothers of sick and preterm infants admitted to a neonatal intensive care unit in Germany. Descriptive statistics were calculated for the System Usability Scale (SUS) and for self-developed items pertaining to overall satisfaction and perceived usefulness of the app. These included items on evidence-based information and the usability of tracking functions.

**Results:**

Of 341 mothers who were contacted, 80 responded (response rate, 23.4%), and data from 63 mothers were analyzed. The mean SUS score was 76.4. The overall satisfaction rate was high, with 84% (n=53) of respondents indicating that they were either satisfied or very satisfied. Further, 82% (n=52) were inclined to recommend the web app to other parents. On average, the evidence-based information was perceived as helpful, more detailed, and not contradictory compared to information provided at the hospital. At the same time, most of the users reported that the Neo-MILK web app did not exert pressure to provide breast milk to their infants. Approximately 71% (n=45) of the mothers used the documentation tool in the web app several times per week to track their milk volumes.

**Conclusions:**

By combining evidence-based information and useful tools to document milk volume, the Neo-MILK web app was high rated in usability and perceived usefulness. Considering the limitations of the study, this web app appears to be a valuable tool for educating and supporting pump-dependent mothers of sick and preterm infants during lactation.

## Introduction

For all infants, mother’s own milk (MOM) should be the first choice of nutrition and is, therefore, recommended by the World Health Organization [1,2]. Its health-promoting effects are evident and sick and preterm infants, in particular, benefit from the nutrition with MOM due to their high vulnerability [3]. However, achieving a sufficient milk volume has shown to be challenging for mothers of infants admitted to a neonatal intensive care unit (NICU) [4]. As many of these mothers are pump-dependent and cannot directly breastfeed, they require structured lactation support to facilitate MOM feeding in their sick and preterm infants [4,5]. This support enables timely communication about the importance of MOM, early initiation of lactation, and frequent pumping to establish a sufficient milk supply, thus ensuring the infants nutrition with MOM [4,6]. When providing lactation support to mothers, sensitive communication without distressing mothers should be facilitated, as stress may negatively affect lactation [7-9]. Indeed, perceived pressure to provide breast milk has been commonly reported among mothers, which may be associated with other mental health issues [10-12].

Mobile health (mHealth) apps and web app technologies are used in various settings such as disease management, medication reminders, and rehabilitation to promote and support a healthy lifestyle [13]. Several mHealth apps focused on breastfeeding already exist, which have shown to improve breastfeeding knowledge, attitudes, and self-efficacy [14-16]. However, mothers with infants admitted to a NICU require special tools and approaches from an app during lactation due to their unique situation. In general, NICU parents report a high demand for information and a willingness to use electronic devices for information and support [17,18]. This potential is further supported by studies which evaluated evidence-based education and information-sharing apps may promote parental mental health, parent-infant relationship, and family-centred care [19-21]. However, while many apps are already available for this target group, a systematic review showed that only a few of them are of good quality [22]. There is a notable lack of evidence-based and scientifically evaluated mHealth technologies focusing on combining evidence-based information sharing with valuable tools for NICU parents throughout the lactation process. The Neo-MILK web app aims to fill this gap for mothers with infants admitted in the NICU, by including both tools such as milk volume tracking and multimedia evidence-based information on several topics (eg, pumping, breastfeeding, or skin-to-skin contact). It was developed as part of the Neo-MILK project, which supports lactating mothers of infants admitted to a NICU [23]. Therefore, the intention of this web app was to complement rather than replace essential personal and direct lactation support provided within NICUs.

To measure the overall usability of mHealth technologies, the system usability scale (SUS) is a widely used instrument, which showed robust results in previous studies [24]. In addition, usability studies often report on ease of use, user experience, and user engagement, depending on the focus of the study [25]. Therefore, the objective of this study was to examine the usability and perceived usefulness of the evidence-based information about lactation and documentation tools contained in the Neo-MILK web app.

## Methods

### Research Design

A quantitative, prospective, and cross-sectional questionnaire was developed to evaluate the usability and perceived usefulness of the Neo-MILK web app.

### Setting and Relevant Context

Participants in this study were mothers of preterm and sick infants admitted to a NICU. In Germany, initiation of lactation is often suboptimal [26]. This may be attributed to the often not implemented structured lactation support and lack of consistent information and personal support during lactation [27]. Mobile health technology may serve as a beneficial tool to help provide additional support to pump-dependent mothers.

### Web App Development

**Figure 1.**
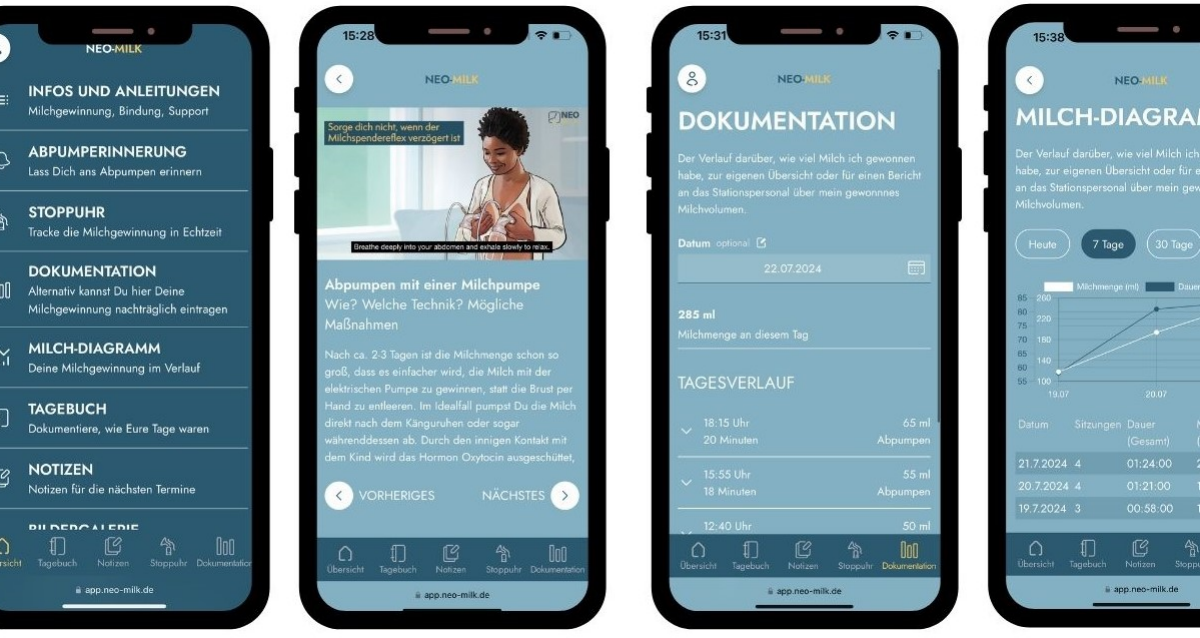
Web app design (left to right: landing page after registration, informational videos, documentation tool, milk diagram).

In a participatory process involving mothers of very low birth weight (VLBW, <1500g) infants, features and content for the Neo-MILK web app were developed by a multidisciplinary team consisting of psychologists, economists, sociologists, neonatologists, neonatal nurses, and a web app developer. To determine the tools and features desired by mothers of VLBW infants, open questions were posted on Instagram. These questions were then ranked in an anonymous online survey completed by 138 mothers. Individual issues, such as the preferred type of communication, were also subject to direct voting via Instagram. Following this participatory process, the final design excluded photographs and instead featured animated parent-infant dyads and drawings. The illustrations were intentionally inclusive to reach a target group which is as diverse as possible. Therefore, the design includes people from different races and ethnicities. The illustrations and texts were developed through a process of various feedback rounds with the multidisciplinary team. As the Neo-MILK web app was part of the Neo-MILK project, the wording and presentation of the web app were aligned to the other materials developed within the project [23]. Finally, tools for recording the time, frequency, and volume of milk during expression, as well as a reminder for milk expression were included. Furthermore, evidence-based information on important topics related to lactation (eg skin-to-skin contact, bonding, milk expression, and hygienic handling of breast milk), along with answers to frequently asked questions regarding breastfeeding and pumping in the form of texts and videos are included in the web app to increase maternal knowledge. To reduce barriers to evidence-based information on pumping and breastfeeding, the videos were featured with subtitles in other languages. These were translated by native speakers working in the Neo-MILK team. Additionally, features such as a diary to document individual milestones of the child and the NICU-related reflections including the mother’s mood and feelings were also provided. In cooperation with a media company (TAKEPART Media + Science GmbH), the app was designed as a browser-based web app (Figure 1).

### Measurements

The overall usability was measured using the SUS, which is a widely accepted tool to indicate usability, operability, and suitability for the use of digital health technologies [28]. Four slightly different translations of this scale are available in German. They were reviewed in a recent publication and revealed that the version by Rummel [29] described the developmental process and methodology most clearly. It was shown to be the best balance between methodologically clean development and wording and, therefore, was utilized in this study [29,30].

To measure the perceived usefulness of the information, videos and the documentation tools, self-developed items were assessed. Overall satisfaction was measured using a question: “How satisfied are you with the web app in general?” with a 4-point Likert scale (“very satisfied”, “satisfied”, “not satisfied”, “not at all satisfied”). Moreover, participants were asked if they would recommend the web app to other parents with the following answering options: “yes”, “rather yes”, “rather no”, and “no”. Perceived usefulness of the information of videos was measured through a 4-point Likert scale of agreement (“agree”, “rather agree”, “rather disagree”, “disagree”) to three statements each for texts and videos which were as follows: (1) “The information I obtained from the texts (videos) were/are helpful for pumping and breastfeeding”, (2) “The information I obtained from the texts (videos) were/are contradictory to those in the hospital,” and (3) “The information I obtained from the texts (videos) were/are more detailed than those in the hospital.”

To assess whether and how frequent mothers used the documentation tool for assessing their pumped milk volume using the following question “How frequently do you use the documentation tool?” which could be rated as: “several times a day”, “daily”, “several times a week”, “several times a month”, “less frequent”, or “never.” Moreover, they were asked whether they found the documentation tools easy to use in a 4-point Likert scale (“difficult”, “rather difficult”, “rather easy”, or “easy”). Furthermore, to analyze whether the Neo-MILK web app caused negative feelings in mothers in terms of pressure to provide breast milk, the following statement was posed: “I felt pressured to provide breast milk by the Neo-MILK web-app” and could be rated on a six-point Likert scale (“totally disagree”, “mostly disagree”, “rather disagree”, “rather agree”, “mostly agree”, “totally agree”). Data on feeding methods as well as previous experience with pumping or breastfeeding, and sociodemographic variables (eg, education, maternal age, infant’s age) were collected. Due to small group sizes, the education level was dichotomized into lower education (lower secondary or secondary school) and higher education (higher education entrance qualification or university degree).

### Data Collection

Data collection took place from September 2023 to May 2024 through the anonymous online survey tool Lime Survey (LimeSurvey GmbH). Data were collected and analyzed by researchers at the University of Cologne, who were also the principal investigators of the Neo-MILK project.

### Data Analysis

Descriptive statistics including mean (SD), depending on the data distribution were calculated. Group comparisons between educational groups concerning the system usability were performed. Figures were used to visualize the data. Missing values were indicated in the description of the baseline characteristics and, in case of figures, in the figure legends. Data were analyzed using STATA (version 18; StataCorp).

### Ethical Considerations

Ethical approval for this study was obtained from the ethics committee of the University of Wuppertal (SK/AE 230329). All methods were performed in accordance with this approval. Before being permitted to complete the questionnaire, the participants were required to consent to the data protection regulations to realize informed consent. The resulting data was anonymous, as no personal data was collected. To compensate the time and effort of the participants, they were offered the opportunity to receive a €10b (US $11.60) voucher for a nationally represented drugstore.

## Results

### Sample

**Figure 2.**
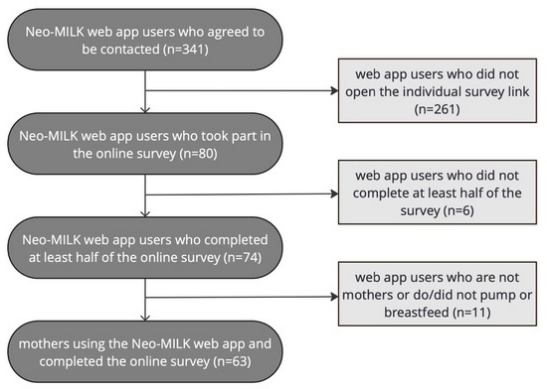
Flowchart of recruitment, inclusion, and exclusion of cases.

Four to eight weeks after registering in the web app, mothers of sick or VLBW infants were invited to participate in the survey. The requirement for registration was explicit consent to be contacted for scientific studies during the registration process. Therefore, 341 mothers received an email with individual tokens to participate in the survey and were reminded once via email if the questionnaire was not submitted within a two-week period. To further ensure correct sampling, all respondents were asked to indicate whether they were currently engaged in pumping or breastfeeding. Of the 341 mothers who were contacted, 80 responded, representing a response rate of 23.4%. Finally, data from 63 mothers were used for statistical analysis (Figure 2).

### Sample Characteristics

Maternal sociodemographic characteristics (eg, age, educational level, native language), and whether reasons for expressing milk stem from maternal or child-related factors, as well as information about their infants (ie, past or current hospitalization, age, birth weight) are presented in Table 1.

**Table 1.** Characteristics of participating mothers and their infants.

Sociodemographic variables	Participants (n=63)
Age^a^ (years), mean (SD)	33.5 (4.6)
Education, n (%)	
Lower secondary/secondary school	14 (22)
Higher education entrance qualification/university degree	48 (76)
Missing data	1 (2)
Native language, n (%)	
Other	4 (6)
German	57 (91)
Missing data	2 (3)
Parity, n (%)	
Singleton	61 (97)
Multiple	2 (3)
Missing data	0 (0)
Infant’s hospitalization (past or currently), n (%)	
No	12 (19)
Yes	51 (81)
Missing data	0 (0)
Infant’s age^b^ (weeks), mean (SD)	8.6 (3.7)
Birth weight (grams), n (%)	
<500	3 (5)
500‐999	15 (24)
1000g-1499	6 (10)
1500-1999	4 (6)
2000‐2500	6 (10)
>2500	4 (6)
Missing data	25 (40)
Reasons for pumping, n (%)	
Maternal-related factors	8 (13)
Child-related factors	49 (78)
Preterm birth	32 (65)
Congenital condition	10 (20)
Birth complications	5 (10)
Others	2 (5)
Missing data	6 (10)

a There were 59 responses for this questions (ie, 4 did not response).

b There were 53 responses for this question (ie, 10 did not response).

### Usability and Perceived Usefulness

**Figure 3.**
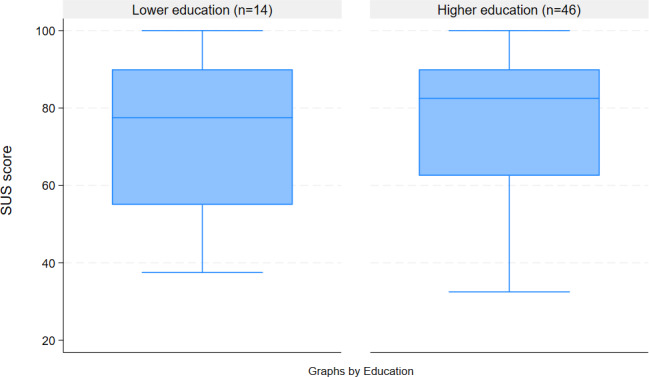
Boxplot of SUS score between the two educational groups. SUS: system usability scale.

The SUS score showed good usability of the Neo-MILK web app, with a mean score of 76.4 (n=61). The score only slightly differed between the lower and higher educational groups (Figure 3).

**Figure 4.**
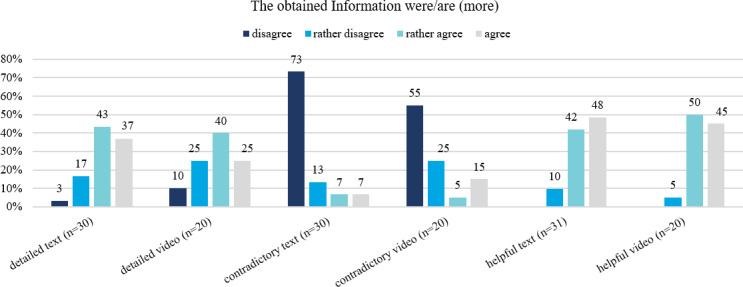
Distribution of agreement whether information in the web app (texts/videos) were more detailed than or contradictory to those in the hospital, or helpful for pumping/breastfeeding.

Overall, 62 participants reported high satisfaction with the web app, with 53 (84%) participants being satisfied or very satisfied. Moreover, 52 (82%) would recommend the web app to other parents. The information provided in the web app via texts were accessed by 31 (49%) and videos by 20 (32%) of the participants. Most participants agreed that the information were more detailed than those provided in the hospital and that they were helpful for pumping and breastfeeding. The majority of participants disagreed that the content was contradictory (Figure 4).

Of the 63 participants, 45 (71%) used the pumping documentation tool of the web app (ie pumping timer or manual documentation of pumping) at least several times a week. Of the 54 participants who rated its ease of use, 46 (85%) found it “rather easy” or “easy to use.”

Of 62 mothers, 56 (89%) indicated that they did not feel pressured by the Neo-MILK web app to provide breast milk, while only 6 (11%) reported experiencing such pressure.

## Discussion

### Principal Findings

This study showed that the Neo-MILK web app provides a suitable tool for mothers of VLBW and sick infants during lactation and pumping. The usability is good, as indicated by SUS scores being clearly over the widely accepted benchmark of 68 [24]. This is further supported by the positively rated overall satisfaction with the web app and the high number of participants who would recommend the app to other parents.

One main feature of the Neo-MILK web app is the provision of evidence-based information about central issues when pumping breast milk for a sick or preterm infant. These include bonding, breast milk expression, and solutions for problems with lactation. Recent studies showed that infant feeding applications are often of poor quality, with incomplete information and lacking in evidence-based content [13,22]. The information provided in the Neo-MILK web app was developed with a multidisciplinary expert team to ensure evidence-based content. Indeed, the results demonstrate that, on average, the information provided was helpful for mothers in their experience of pumping and breastfeeding. Furthermore, the information was more detailed than that provided in the hospital and not contradictory. It can therefore be assumed that the Neo-MILK web app is a suitable tool for informing mothers with an infant admitted to a NICU. However, it remains unknown, whether the mothers in our study received any personal lactation support or if the Neo-MILK web app was the only source of information and support available to them. It is important to emphasize that this web app should provide additional features and information for mothers. Personal and direct lactation support in the NICU, as part of a MOM-friendly hospital policy is crucial for ensuring nutrition with MOM [31,32].

Another central tool is the option to document the expressed milk volume. A recent study showed that tracking features lead to higher ratings of breastfeeding applications in general [33]. This indicates its importance for mothers and may be even more notable for pump-dependent mothers who are not able to feed their infant on demand. In our study, this assumption is strengthened, as most participants used the documentation tools to track their milk volume. In addition, the ranking of the ease of use indicates good usability of these features. This is particularly relevant, as usability has shown to be a crucial factor for the adoption of health technologies [34].

As elaborated before, stress and lactation are assumed to be associated [7,8]. This association is further assumed to be bidirectional, with stress leading to negative breastfeeding outcomes and impaired lactation increasing stress [35]. Due to the high levels of stress NICU mothers perceive, it is of paramount importance to minimize stress rather than triggering further distress [9]. Recent studies showed that mothers feel specific pressure to breastfeed or provide breast milk, which can stem from internal or external factors, for example, from their social environment or health care providers [11,12,36]. However, even though the Neo-MILK web app contains a lot of information about the importance of MOM, as well as tracking options for milk supply, users do not perceive these features as triggering such feelings.

### Limitations

This study has some limitations. The SUS is available in four German translations, all of which have different deficiencies, for example, in methodology or wording. Although there is a validated version available, it lacks in terms of translation, leading to unnatural wording. In this analysis, we used the recommended translation, which is regarded as the best compromise between methodologically rigour and comprehensibility [30]. This might have had an impact on the validity of the measurement and therefore influenced the results. The response rate of this study is low. It may be the case that the users were no longer using the web app at the time of the survey. This may particularly apply to mothers who have transitioned from pumping to breastfeeding after a few weeks, and therefore no longer require the tools of the Neo-MILK web app. Another reason for the low response rate could be that registered web app users who were unsatisfied or were not frequent users did not respond due to their dissatisfaction. Furthermore, it can be suggested that the utilization of mHealth technologies is determined by the educational level and age of users, which may indicate a healthy bias among its users [37]. This is reflected by the broad number of participants with a high educational level in this sample. However, the Neo-MILK web app utilization and its ratings might not be biased by educational level, as indicated by only slight differences in the SUS score between these groups. Nevertheless, the information should be accessible for all groups to reduce communication difficulties, including those due to language barriers, which are a common problem in health services [38]. To reduce these barriers, the videos in the Neo-MILK web app are available with subtitles in other languages, including Russian, French, English, and Turkish. In addition, the videos are composed of easily understandable images and keywords. However, the provision of a translated version of the entire web app when necessary would further facilitate the accessibility of the web app to all relevant target groups.

### Conclusions

The combination of evidence-based information about lactation and useful tools for tracking was identified as being of particular importance for this special group of mothers, with both elements rated highly in terms of usability and usefulness. Therefore, it can be concluded, that in our study, the Neo-MILK web app serves as a useful tool to complement personal support for pump-dependent mothers during lactation.

## Abbreviations

mHealth: mobile health
MOM: mother’s own milk
NICU: neonatal intensive care unit
SUS: system usability scale
VLBW: very low birth weight
